# Coordinated Development of Population, Resources, Environment, Economy, and Society under Engineering Management Combined with Bilevel Optimization Model

**DOI:** 10.1155/2022/8589396

**Published:** 2022-01-15

**Authors:** Kai Chen, Yilin Chen

**Affiliations:** School of Economics and Trade, Northeastern University at Qinhuangdao, Qinhuangdao 066000, China

## Abstract

The in-depth analysis of the strategies for the coordinated and continuous development of population, resources, environment, economy, and society based on the engineering management model is highly important for the sustainable development of the regional economy and society. In this article, a population-economy-resources-environment bilevel optimization model is established based on the economic and social development in a provincial region. The method of bilevel optimization is adopted to introduce the specific bilevel optimization model. The concept and objectives of the bilevel optimization are explained, and its corresponding technical applications are described. In this article, the development in coordinated economic and social development of population, resources, and environment is analyzed and compared based on the bilevel optimization model. In particular, the evolution and changes before and after the implementation of engineering management are studied. Through the results, it can be observed that after the implementation of project management, the coefficient of industry location has presented a downward trend, and the coordinated development of population, resources, environment, economy, and society has become more coordinated.

## 1. Introduction

With the rapid economic development in recent years, the social resources acquired are getting greater and greater. As a result, it is impossible to sort out these resources based on the previous means and techniques [1]. In this context, the bilevel optimization model has become an important component in the coordinated economic and social development of population, resources, environment, with a low threshold and strong convergence characteristics possessing advantages [2, 3]. In the 21st century, people become more demanding for the relationships in the coordinated development of population, resources, environment, economy, and society. The previous simple economic growth can no longer meet their needs, and the coordinated development of population, resources, environment, economy, and society has slowly shifted from a single nature into an interactive field [4, 5]. The coordinated development functions allow people to identify useful resources among massive resources. Hence, the combination of the coordinated development function and the coordinated economic and social development of population, resources, environment, and society are useful, and the combination of these two techniques can facilitate a better management of the coordinated economic and social development of population, resources, and environment.

The application of the bilevel optimization model can explain the previous concept of spatial location of production more effectively, while providing the corresponding way to new trade concepts and newly added concepts. After the analysis and evaluation of the basic concepts and techniques of the bilevel optimization model, a special study of the concept is conducted in this article on the situation of regional development in China. Through the study, it is concluded that the bilevel optimization model has played a crucial role in promoting the coordinated development of population-resource-environment-economy-society regarding the differences in regional development. Finally, this article provides a theoretical basis for the future coordinated development degree of population-economy-resource-environment system in the province and puts forward a strategy to promote the coordinated development of socio-economic and resource-environment scientifically and rationally, so as to provide a theoretical basis for the sustainable development of economy and environment in the province.

## 2. Bilevel Optimization Model

In this article, the bilevel optimization model is introduced, and it can be observed that the initialization of the bilevel optimization center can have a serious effect on the bilevel optimization model if the results are not reasonable. This effect requires massive computation time for engineering management under the population, resources, environment, economic, and social subjects intelligent search engine. At the same time, it will also greatly reduce the performance of bilevel optimization, which will eventually lead to inaccurate results obtained.

**Figure 1:**
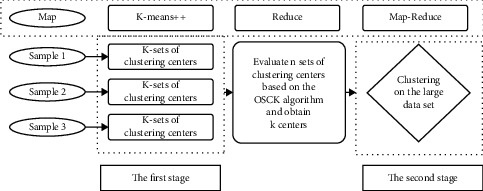
Framework of the method proposed in this paper at two stages.

Based on the working principle of the bilevel optimization model cited in this paper, the method of small-sized samples and multiple sampling are dopated. Through the bilevel optimization model, the scientific and reasonable bilevel optimization center can be obtained so that the initialization stage of the bilevel optimization unreasonable phenomenon can be eliminated based on the big data bilevel optimization model to effectively improve the performance and accuracy of big data bilevel optimization model. The core idea of the model is based on small sample collection, followed by the bilevel optimization model analysis based on the Map-reduce model. The operational framework of the bilevel optimization model is shown in Figure 1.

In the next section, the optimization of the bilevel optimization model *K*-value is described in detail. The model consists of three main parts: separation model, subcenter elimination, and weight-*K* value optimization model.

It is assumed that that *s* stands for the number of samples, *x* stands for the coordinates, *i* stands for the ith sample, *k* stands for the number of clusters, and *d* stands for the data dimension, the result set of the ith sample cluster is represented in the following form:

(1) $$\begin{array}{c} \left(x_{11}^{i},x_{12}^{i},\ldots ,x_{1\;d}^{i}\right)\left(x_{21}^{i},x_{22}^{i},\ldots ,x_{2\;d}^{i}\right)\cdots \left(x_{k1}^{i},x_{k2}^{i},\ldots ,x_{k\;d}^{i}\right). \end{array}$$

The matrix form of the results obtained based on the bilevel optimization for all samples is shown as follows:

(2) $$\begin{array}{c} \left[\begin{array}{cccc} \left(x_{11}^{1},x_{12}^{1},\ldots ,x_{1\;d}^{1}\right) & \left(x_{21}^{1},x_{22}^{1},\ldots ,x_{2\;d}^{1}\right) & \cdots & \left(x_{k1}^{1},x_{k2}^{1},\ldots ,x_{k\;d}^{1}\right)\\ \left(x_{11}^{2},x_{12}^{2},\ldots ,x_{1\;d}^{2}\right) & \left(x_{21}^{2},x_{22}^{2},\ldots ,x_{2\;d}^{2}\right) & \cdots & \left(x_{k1}^{2},x_{k2}^{2},\ldots ,x_{k\;d}^{2}\right)\\ \vdots & \vdots & \cdots & \vdots \\ \left(x_{11}^{s},x_{12}^{s},\ldots ,x_{1\;d}^{s}\right) & \left(x_{21}^{s},x_{22}^{s},\ldots ,x_{2\;d}^{s}\right) & \cdots & \left(x_{k1}^{s},x_{k2}^{s},\ldots ,x_{k\;d}^{s}\right) \end{array}\right]. \end{array}$$

In the above matrix, the arrangement of matrix elements corresponds one to the other based on the similarity of cluster centers. A column matrix corresponds to clusters, in which *k* clusters are shared. It is assumed that the kth cluster center of the first sample, the first cluster center of the second sample, and the second cluster center of the last sample are subcenters.

(3) $$\begin{array}{c} \left[\begin{array}{cccc} \left(x_{11}^{1},x_{12}^{1},\ldots ,x_{1\;d}^{1}\right) & \left(x_{21}^{1},x_{22}^{1},\ldots ,x_{2\;d}^{1}\right) & \cdots & \left(x_{k1}^{1},x_{k2}^{1},\ldots ,x_{k\;d}^{1}\right)\\ \left(x_{11}^{2},x_{12}^{2},\ldots ,x_{1\;d}^{2}\right) & \left(x_{21}^{2},x_{22}^{2},\ldots ,x_{2\;d}^{2}\right) & \cdots & \left(x_{k1}^{2},x_{k2}^{2},\ldots ,x_{k\;d}^{2}\right)\\ \vdots & \vdots & \cdots & \vdots \\ \left(x_{11}^{s},x_{12}^{s},\ldots ,x_{1\;d}^{s}\right) & \left(x_{21}^{s},x_{22}^{s},\ldots ,x_{2\;d}^{s}\right) & \cdots & \left(x_{k1}^{s},x_{k2}^{s},\ldots ,x_{k\;d}^{s}\right) \end{array}\right]\\ ⇓\\ \left[\begin{array}{cccc} \left(x_{11}^{1},x_{12}^{1},\ldots ,x_{1\;d}^{1}\right) & \left(x_{21}^{1},x_{22}^{1},\ldots ,x_{2\;d}^{1}\right) & \cdots & \left(0,0,\ldots ,0\right)\\ \left(0,0,\cdots ,0\right) & \left(x_{21}^{2},x_{22}^{2},\cdots ,x_{2\;d}^{2}\right) & \cdots & \left(x_{k1}^{2},x_{k2}^{2},\ldots ,x_{k\;d}^{2}\right)\\ \vdots & \vdots & \cdots & \vdots \\ \left(x_{11}^{s},x_{12}^{s},\ldots ,x_{1\;d}^{s}\right) & \left(0,0,\ldots ,0\right) & \cdots & \left(x_{k1}^{s},x_{k2}^{s},\ldots ,x_{k\;d}^{s}\right) \end{array}\right]. \end{array}$$

In the above matrix (0, 0, ..., 0), which is the bilevel optimization center, is shown as a subcenter. In fact, it is still unknown what cluster centers are suboptimal. Hence, the next topic is to locate and remove the following suboptimal cluster centers.

## 3. Overview of the Study Area

**Figure 2:**
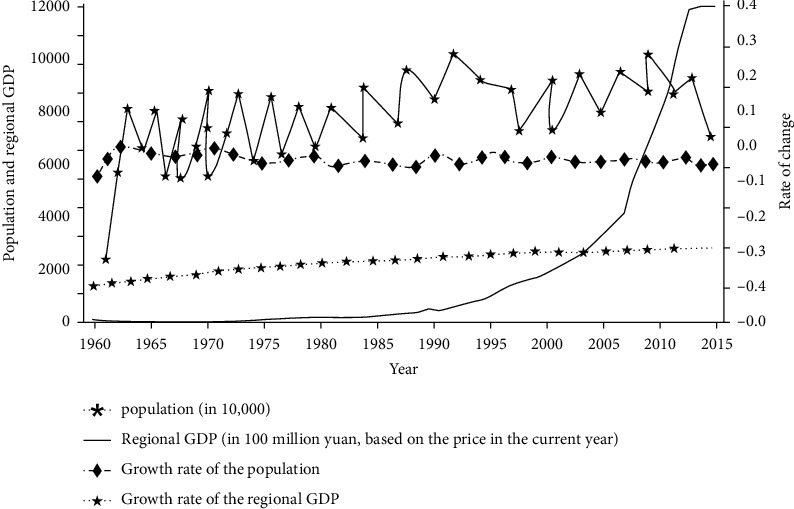
Diagram of demographic and economic development trends in the region.

The population of the province has shown an increasing trend since 1990, and the population growth rate has remained relatively stable, with a small downward trend during this period. The total production in the region of the province is characterized by obvious stage, presenting a gradual trend of growth in the fluctuating gross product of the province in the period from 1960 to 1980. In the period from 1981 to 2000, the gross production in the region showed a trend of annual growth, and in 1995, the gross production in the region exceeded 100 billion yuan with fluctuations. In the period from 2001 to 2014 with the implementation of the policy of revitalization of the old industrial bases in the region, the GDP showed significant growth characteristics until 2014 when the GDP reached RMB 1380.44 billion at the same time, the growth of the regional GDP was maintained at about 0.14%, and the regional economic growth gradually showed a growth trend (Figure 2).

The region is in a period of industrialization transformation and accelerating urbanization. The share of the three industrial structures in the region in 2014 was 11.1%, 52.7%, and 36.2%, respectively. Among them, the value of the primary industry in the regional industrial structure grew RMB 15.457 billion, corresponding to a growth rate of 4.7%. The growth value of the level 2 industry in the region is RMB 728.727 billion, with a corresponding growth rate of 6.7%, and the growth value of the tertiary industry in the region is RMB 499.198 billion, with a corresponding growth rate of 6.8%. The rapid development of industrialization and urbanization will lead to an increase in energy consumption in the city, from which it can be seen that in 2014 the energy consumption in the region was as high as 84.835 million tons, corresponding to a coal consumption rate of 72.5%, which is far above the national average. The majority of the end-use energy consumption is concentrated in the level 2 industry, mainly in coal consumption, while oil, electricity, and heat consumption are also relatively large.

## 4. Research Method and Data Sources

### 4.1. Regional Population-Economy-Resources-Environment Scheduling Forecast Model

The dynamics and complexity of the coordinated development of population, economy, and resources and environment in this region cannot be predicted based on a single conventional linear or nonlinear model of its future development trends. In this article, the parameters in the evaluation indexes are optimized by using a bilevel optimization model based on the scheduling and evaluation system of the population-economy-resource-environment association.

#### 4.1.1. Establishment of the Index System and Determination of the Weights

In this article, the development evaluation index system of this region is mainly established with the goal of population-economy-resource-environment coordination, and the division of population development, economic development and resource-environment development is carried out based on bilevel optimization model. The corresponding indexes are combined by qualitative and quantitative methods, as shown in Table 1.

#### 4.1.2. Coordination Degree Model

Coupling refers to the synergistic phenomenon due to the interaction and mutual influence between two or more systems and components, and the degree of bonding is a quantitative description of the degree of interaction and influence between systems and components. The synergistic forecast model for the population, economy, and resource environment established in this paper includes two parts: objective function and constraint conditions.

(1) Objective function*:* based on the concept of capacitive coupling in physics and the capacitive coupling coefficient model, the coordination degree model of population-economy-resource-environment (3-system) interaction is promoted.

(4) $$\begin{array}{c} D={\left(C\times T\right)}^{1/2}. \end{array}$$

In the above equation, *D* stands for the degree of coordinated development, which is [0, 1]. *c* stands for the degree of coupling, which reflects the level of synergy between the interactions of systems. *T* stands for the harmony index, which reflects the overall cooperative effect or contribution of population, economy, and resource environment. C and *T* can be expressed as follows:

(5) $$\begin{array}{c} \left\{\begin{array}{c} C={\left\{\frac{f\left(x\right)\cdot g\left(y\right)\cdot h\left(z\right)}{\left[f\left(x\right)+g\left(y\right)\right]\cdot \left[f\left(x\right)+h\left(z\right)\right]\cdot \left[g\left(y\right)+h\left(z\right)\right]}\right\}}^{1/3},\\ T=\alpha \cdot f\left(x\right)+\beta \cdot g\left(y\right)+\gamma \cdot h\left(z\right). \end{array}\right. \end{array}$$

In the above equation, the candidate quantities *α*, *β*, *γ* of the population-economic-resource-environment subsystem are finally set in this paper after the review of literature and the consultation of experts. *α* = 0.3, *β* = 0.2, *γ* = 0.5; *f*(*x*) is a function of population effect; *g*(*y*) is a function of economic effect; and *h*(*z*) is a function of resource-environment effect, which are shown in the following, respectively.

(6) $$\begin{array}{c} f\left(x\right)=\overset{m}{\underset{i=1}{\sum }}a_{i}x_{i}^{\prime },\\ g\left(y\right)=\overset{n}{\underset{j=1}{\sum }}b_{j}y_{j}^{\prime },\\ h\left(z\right)=\overset{q}{\underset{k=1}{\sum }}c_{k}z_{k}^{\prime }. \end{array}$$

In the above equation, *x*_*i*_′, *y*_*j*_′, and *z*_*k*_′ stand for positive numbers of specific indexes that represent the demographic-economic-resource-environmental characteristics, respectively. The weight of each index. The extreme value method is used for criterialization. The specific methods are described as follows:

(7) $$\begin{array}{c} x_{i}^{\prime }=\left\{\begin{array}{cc} \frac{x_{i}-\lambda_{\min}}{\lambda_{\max}-\lambda_{\min}}, & \lambda_{\min}\leq x_{i}\leq \lambda_{\max},\;x_{i}\text{ is a positive index}\left(i=1,2,\right),\\ \frac{\lambda_{\max}-x_{i}}{\lambda_{\max}-\lambda_{\min}}, & \lambda_{\min}\leq x_{i}\leq \lambda_{\max},\;x_{i}\text{ is a positive index}\left(i=1,2,\right). \end{array}\right. \end{array}$$

The original data processing method for *y*_*j*_′ based on the above method is *j* = 1 2,3,4,*k* = 1,2,3,4,5,6.

(2) Composition of constraint conditions: first, about the economic development constraints, the regional economic development status can be expressed as follows:

(8) $$\begin{array}{c} {\text{GDP}}_{t}={\text{GDP}}_{t-1}\left(1+k_{\text{GDP}}\right),\\ {\text{GDP}}_{rj_{t}}={\text{GDP}}_{rj_{t-1}}\left(1+k_{{\text{GDP}}_{rj}}\right),\;{\text{GDP}}_{rj}\geq A_{{\text{GDP}}_{rj}}. \end{array}$$

In the above equation, GDP _t_ stands for the regional GDP in year *t*, GDP _t-1_ stands for the regional GDP in year t-1, and *k*_GDP_ is the growth rate of regional GDP. With regard to GDP per capita in year *t*, *k*_GDP_*rj*__ is the growth rate of GDP per capita, and*A*_GDP_*rj*__ is the minimum GDP per capita required for the province.

Emission constraint of environmental pollutants: *W*_*t*_=*W*_*t*−1_(1+*k*_*W*_), *W*_*t*_ ≤ *W*_*t*−1_, *W*_*t*_ ≤ *B*_*t*_, in which *W*_*t*_ is the pollutant (SO_2_/CO_2_) emission in year *t*, *W*_*t*−1_ is the pollutant (SO_2_/CO_2_) emission in year t-1, *k*_*W*_ is the pollutant (SO_2_/CO_2_) emission growth rate, and *B*_*t*_ is the allowed pollutant emission in region in year *t* [4, 6].

Carbon emission intensity: *E*_*t*_=*E*_*t*−1_(1+*k*_*E*_), *E*_*t*_ ≤ *E*_*t*−1_, in which *E*_*t*_ is the carbon emission intensity in year *t*, *E*_*t*−1_ is the carbon emission intensity in year t-1, and *k*_*E*_ is the carbon emission intensity growth rate.

Forest coverage rate: *F*_*t*_=*F*_*t*−1_(1+*k*_*F*_), *F*_*t*_ ≥ *F*_*t*−1_, in which *F*_*t*_ is the forest coverage in year *t*, *F*_*t*−1_ is the forest coverage in year t-1, *k*_*F*_ is the forest coverage growth rate, through ecological restoration and environmental protection and other measures, the regional forest cover in year *t* ≥ the regional forest coverage in year t-1.

(3) Division of the determination criteria for coordination degree: with regard to 3 major categories and 10 subcategories in the coordinated development of population, economy, and resources and environment association schedule, the transitional coordination, dysfunction and decline, each class based on the size of the population, economy, resources, and environment effect function is the population lag type, economic lag type, energy and environmental lag type, synchronous type four types (the details are shown in Table 2).

(4) Settings of the stages and index parameters: based on the current situation of the economy and society and the future development direction of resources and environment in the province, three stage schemes are established in this paper, that is, the standard stage, the stabilization stage, and the coordination stage, respectively.

1. Baseline stage: rapid development of population and economy, with moderate improvement in the resources and environment
2. On the basis of the socioeconomic development in the 11th Five-Year Plan, the population regional production, rapid development of urbanization rate, industry and industry structure has a strong dependence on the level 2 industry coal, oil ratio also remains at a very high level. At this stage, the improvement in the energy efficiency has basically reflected the state of naturally oriented economic development and energy consumption, and it is relatively weak in the implementation of energy conservation and emission reduction measures. Environmental pollutants have yet to be controlled effectively.
3. Robust development of population and economy, with a stable improvement of resources and environment: based on the standard stage, the growth rate of economy, population, and urbanization is slowed down, the reliance on industry and traditional primary energy is reduced, and energy-saving and low-carbon technologies are used to optimize the energy consumption structure and accomplish the goal of energy conservation and emission reduction. In this situation, the implementation of relevant energy conservation and emission reduction measures seeks to balance economic development and environmental improvement, which can basically reflect the state of economic development and resources and environment to be achieved by energy conservation.
4. The coordination stage is highly similar to the stable development of population and economy, with the rapid improvement of resources and environment.
5. This stage is a comprehensive control stage, in which energy saving and emission reduction activities are carried out in the whole society, and the production and lifestyle of the population is comprehensively optimized. In addition, the economic development pattern and consumption pattern of the residents are improved. The energy structure and industrial structure are further optimized, and major breakthroughs are made in energy conservation and emission reduction measures and economic technologies on all fronts [7, 8]. This is basically an active effort to achieve the goal of economic development and the state of resources and environment.

Based on the above description, 12 index parameters such as population, urbanization rate, total regional production, GDP per capita, level 2 production ratio, tertiary production ratio, energy intensity, and nonfossil energy ratio are parameterized as stage elements (the details are shown in Table 3).

(1) Evaluation index system: based on the actual situation of the economic and environmental development and analytical modeling requirements of the county, the index decision follows the following principles: (1) Comparison of indexes is feasible; (2) identification of indexes is significant in the aspect of significance and differences; (3) indexes are highly independent of each other; and (4) the feasibility of index data collection. Based on the above principles, spatial variable analysis and correlation analysis were used to screen the indexes, as shown in the following equation:
  (9) $$\begin{array}{c} C_{ij}=\frac{S_{j}}{X_{j}},\;\left(j=1,2,\ldots ,20\right). \end{array}$$
In the above equation, *C*_*ij*_ stands for the coefficient of variation; *S*_*j*_ stands for the criteria deviation; and *X*_*j*_ stands for the mean value.
Based on equation (1) to determine the raw data belonging to the relevant indexes of each district and county, in addition to the indexes with large correlation coefficients, small spatial variation and identification of meaningful differences, the final 8 indexes that can reflect the economic environment and social interrelationship in the county. These are economic index system, environmental index system, and social development index system [9, 10]. The economic indexes include total industrial production (*X*1), gross domestic product per capita (*X*2), and GDP growth rate (*X*3). The environmental indexes include chemical oxygen demand emissions (*X*4), energy consumption per unit of GDP (*X*5), and pollutant emissions per unit of GDP (*X*6). The social development indexes include rural Engel coefficient (*X*7) and unemployment rate (%) (*X*8).
(2) Determination of the weighting of evaluation indexes: upon the determination of the weighting of evaluation indexes, there will be increasing methods of subjective decision of weighting, such as the AHP method. The evaluation results are sometimes biased based on the subjective factors of human. Based on the information theory, the entropy value reflects the degree of disorder of information, the smaller its value, the smaller the disorder of the system, the greater the utility value of information; the larger that value is, the higher the disorder of the system and the smaller the utility value of the information.
For the initial matrix of *n* evaluation indexes for the *m* programs discussed in the above section, as the determination matrix is obviously an information carrier, the orderliness of the system information obtained through information entropy evaluation and its effects are used to determine the index weights eliminating the artificial interference of the weighting calculation if possible so that the evaluation results can be more practically consistent [11]. The calculation sequence is described as follows:
  (1) The judgment matrix is established based on *n* evaluation indexes for *m* objects as follows:
    (10) $$\begin{array}{c} R={\left(X_{j}\right)}_{nm},\;\left(i=1,2,\ldots ,n;j=1,2,\ldots ,m\right). \end{array}$$
  (2) The decision matrix is normalized to obtain the normalized decision matrix as follows:
    (11) $$\begin{array}{c} b_{j}=\frac{X_{ij}-X_{\text{min}}}{X_{\text{max}}-X_{\text{min}}}. \end{array}$$
  In the above equation, each item is the most satisfied or the most dissatisfied under the same index (the smaller the better), that is, *X*_max_*X*_min_.
  (3) Based on the definition of entropy, *n* evaluation index for *m* evaluation objects is the entropy of evaluation indexes as follows:
    (12) $$\begin{array}{c} \text{Hi}=\frac{1}{\ln\;m}\sum_{j=1}^{m}f_{ij}\ln\;f_{ij}. \end{array}$$
  In the above equation, *f*_*ij*_=*b*_*ij*_/∑_*j*=1_^*m*^*b*_*ij*_. When *f*_*j*_=1, *f*_*j*_ln  *f*_*j*_, different from the degree of disorder of information apparently reflected in the entropy, it is equal to 0 and does not match the actual situation. Hence, *f*_*ij*_ is further modified as follows
    (13) $$\begin{array}{c} f_{ij}=\frac{1+b_{ij}}{\sum_{j=1}^{m}1+b_{ij}}. \end{array}$$
  (4) The entropy weight of the evaluation index is calculated, which is in line with the actual situation.
(3) Establishment of the gray correlation model: for the convenience of analysis, the correlation model is established as follows:
  (1) The optimal vector of the series is determined. As the original data are normalized and transformed into positive indexes, the optimal vector is obtained as follows:
    (14) $$\begin{array}{c} G=\left(g_{1},g_{2},\ldots ,g_{n}\right)=\left(f_{11}vf_{12}v\cdots vf_{1m},f_{21}vf_{22}v\cdots vf_{2m},\ldots ,f_{n1}vf_{n2}v\cdots vf_{nm}\right). \end{array}$$
  In the above equation, *v* is the maximum operator.
  (2) The gray correlation coefficient equation is used to calculate the correlation coefficient between the *j*th evaluation index and the optimal vector *G*, as shown in the following equation:
    (15) $$\begin{array}{c} \xi_{i}\left(Y_{i},G\right)=\frac{\underset{i}{\text{min}}\underset{j}{\min}\left|f_{ij}-g_{i}\right|+\rho \underset{i}{\text{max}}\underset{j}{\text{max}}\left|f_{ij}-g_{i}\right|}{\left|f_{ij}-g_{i}\right|+\rho \underset{i}{\max}\underset{j}{\max}\left|f_{ij}-g_{i}\right|}. \end{array}$$
  In the above equation, the minimum difference between the two poles and the maximum difference between the two poles *ρ*-the decomposition coefficient is generally 0.5; that is, $\underset{i}{\min}\underset{j}{\min}\left|f_{ij}-g_{i}\right|\underset{i}{\max}\underset{j}{\max}\left|f_{ij}-g_{i}\right|$.
  (3) The correlation *R* between the *i*th evaluation object *b* and the optimal vector *G* is calculated, as shown in the following:
    (16) $$\begin{array}{c} R=\sum_{j=1}^{n}w_{j}\times \xi_{i}\left(Y_{i},G\right). \end{array}$$
(4) Output of the results: based on the economic environment data of the county in Shaanxi Province from 2000 to 2009, the original data of the indexes are processed dimensionless using official (2) and (3) to obtain the criteria data of the indexes. The entropy value assignment method is used to conduct calculation based on equations (12) and (13), in which the weight of each index is shown as follows: *W*_*j*_=(0.114, 0.117, 0.123, 0.144, 0.128, 0.128, 0.140, 0.105).

**Figure 3:**
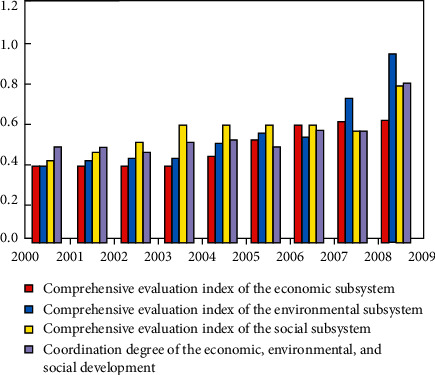
Evolution of the economic-environmental-social system coordination index values in the county.

Based on the normalized data, the series optimal vector *G* = (1,1,1,1,1,1,1,1,1) tab is obtained. The above correlation coefficients are introduced into equation (16), and the correlation coefficient F is introduced into equation (15) to derive the status of the passing of the values of the coordination index of the economic, environmental, the social system subsystems, and the composite system from 2000 to 2009 (Figure 3).

## 5. Data Sources

The parameter values of evaluation indexes in this paper are set in accordance with the relevant literature such as the Statistical Yearbook of the Province, the economic and social development goals in the 12th Five-Year Plan, and the historical development experience of world economies.

### 5.1. Analysis of Results and Discussion

Based on the analysis of the overall development trend in this region, the growth trend in the standard stage and the stability stage from 2011 to 2030 presented a “*U*-shaped” trend. However, in the coordination stage, it mainly shows a development trend of increases year by year.

### 5.2. Analysis of Changes in the Time Series of the Coordinated Development Degree

**Figure 4:**
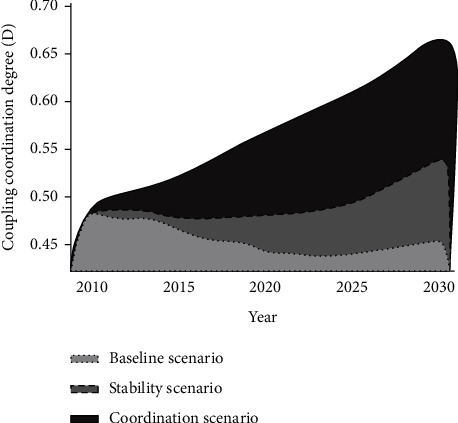
Changes in the time series of the coordination development degree at different stages: stages of population-economy-resources-environment scheduling and comparative analysis.

With the gradual improvement of the economic level in the region, the level of science and technology has also been developed in an unprecedented speed. In addition, the optimization of energy structure and energy utilization rate is also increasing year by year. In the situation of the coordinated development of the population-economy-resources-environment system, the growth of the regional economy in the standard stage and the stable stage has been improving from the historical low to zero in about 2030. Through comparison with the standard stage and the stable stage, the adjustment stage focuses more on energy saving and emission reduction activities in the whole society and proactively implements the rapid improvement of resources and environment through major breakthroughs in policy, economy, and technology in all aspects, and the degree of coordinated development has been maintaining an upward tendency [12, 13]. By 2030, the growth rate will be 0.64 (basically coordinated), which is 1.4 times that in 2010. The current stage represents the ideal model for the coordinated development of the regional population, economy, resources, and environment in this province (Figure 4).

#### 5.2.1. Baseline Stage

**Figure 5:**
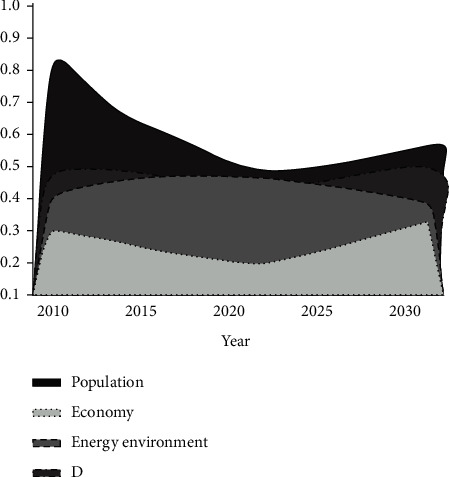
Changes in the coordination degree at the baseline stage.

The province is now in a period of steady urbanization. The population keep growing, with an average annual growth rate of about 0.2% and a declining population efficiency function (the details are shown in Figure 5). The economic development model at present is indispensable for the rapid development of industrialization. Under this background, the ratio of level 2 sector in the province is increasing with an average annual growth rate of about 0.6% industrial structure depends on level 2 sector. The economic efficiency is significantly lagging behind. Population and resource environment effects are not obvious. Until 2020, with the implementation of measures such as the improvement of energy use efficiency and nonfossil energy ratio, the emissions of SO_2_ have been effectively suppressed with an annual average reduction of -1.14%. With this effect, the resource and environmental effect function increases slightly, reaching a peak in 2019. However, with the economic and social development, CO_2_ emissions increased significantly and offset the resource-environmental effect brought by the reduction of SO_2_ emissions. Hence, the resource-environmental effect has been decreasing year by year subsequently.

Based on the analysis in the above sections, the economic development and energy consumption in the region are coordinated based on the baseline scenario development, which has ultimately kept the regional development in a low state, and the corresponding socioeconomic growth pattern is an extensive form. However, with the extension of time, the progress of resource and environment improvement lags far behind the economic and social growth rate, which makes the resource and environment growth efficiency unable to support, ultimately become a hindrance to the coordinated development of population-economy-resource and environment, and have a negative impact on the sustainable development of the whole society in the region.

#### 5.2.2. Stability Stage

**Figure 6:**
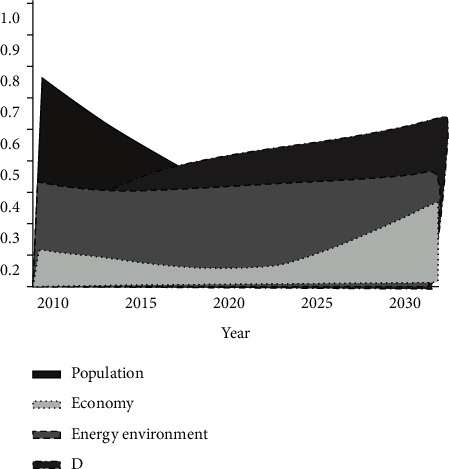
Changes in the coordination degree at the stability stage.

In the stability growth stage of the region, the population growth trend presents a U-shaped trend of gradual development, as shown in Figure 6. The urbanization rate can be effectively controlled to some extent, and the population growth function can achieve a level lower than the reference value to some extent. The industrial structure can be effectively adjusted. Based on the economic growth of GDP, GDP per capita and the proportion of tertiary industry show an increasing trend every year. The economic growth trend achieved the minimum level of 0.242% in 2020 and started to increase year by year in the later period [14, 15]. The utilization rate of nonfossil energy will be effectively improved, and the growth rate of pollutant emission will be suppressed. The annual average growth rate of SO_2_ and CO_2_ emission from 2010 to 2020 was reduced from −80% to 2.6%, which was a decrease of 20.5% and 37.2%, respectively, compared with the standard stage, with a more significant effect on resources and environment. With the joint effect of population, economic, and resource-environmental effect functions, the stability stage of coordinated development degree has the same tendency of change as the baseline stage, with a U-shaped tendency of decreasing first and then increasing. Timing and size of its trough tips are significantly different from those in the baseline stage. Different from the criteria scenario, with the economic benefits of stable development, the value of the trough in 2020 was 0.478. However, by 2027, the value of development within the region will increase to 0.511, after which the development of the region will start to enter the stage of uncoordinated development, when the economic growth rate hits the lowest level among the three. This stage between 2010 and 2026 is on the verge of stagnation in the economic development.

#### 5.2.3. Coordinated Development Stage

**Figure 7:**
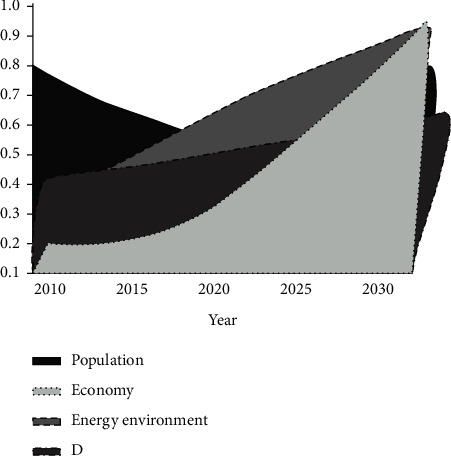
Changes in the coordination degree at the coordination stage.

Through the overall optimization of production, change of lifestyle, and industrial structure in this region, the effective control of population growth in the region is carried out multiple times. In addition, under the premise of effective control while achieving the increase of urbanization level at the same time, the trend of population growth from 2010 to 2030 does not present a valley phenomenon. However, with the implementation of the resource and environmental improvement policies, the average annual growth rate of the emission of SO_2_ and CO_2_ is maintained at about -1.1%; and with the significant control of pollutant emissions, the energy efficiency function can always keep a significant growth trend, with the robust improvement in the annual growth. In 2027, the economic efficiency in this region is the lowest among the three. However, in 2028 and 2030, the demographic effect and resource environment were eliminated. Hence, the development was the fastest among the three. From 2024 (0.0.6%) to 2026 (0.622%), it will be the period of relatively backward economic development; from 2027 to 2029, it will be the primary coordinated development with backward population, and 2030 will start to enter the primary coordinated development period with stagnant growth in the resources and environment (Figure 7).

Based on the trend diagram in Figure 7, the coordinated stage development phase occurs at different stages of production effects and profit lag can be obtained. However, as the development trend of population, economy, and resources and environment in the region gradually approaches 1, the evaluation indicates that the overall situation gradually moves towards coordinated development and eventually enters the model of coordinated development of population, economy, resources, and environment.

## 6. Conclusions

In this article, a bilevel optimization model is used to establish an evaluation system for the coordinated development of population, economy, resources, and environment within the region, and the established evaluation index system is used to carry out accurate forecast for the economic development within this region. Through the experimental analysis, it can be known that for the purpose of achieving the coordinated development of population, economy, resources, and environment in this region, the effective regulation of urbanization development level should be carried out in the aspect of population growth. In the aspect of economic benefits, it is necessary to accelerate the speed of economic transformation in the region and optimize the industrial structure. In the aspect of resources and environment, it is necessary to develop and utilize nonfossil energy effectively and fully improve the utilization rate of resources and energy. In this way, the coordinated and rapid development among population, economy, resources, and environment in the region can be achieved gradually.

## Figures and Tables

**Table 1:** Scheduling evaluation index system and weights of the population-economy-resource-environment association in the province.

Target level	Criteria level	Index level	Nature of index	Weight
Comprehensive development level of population, economy, resources, and environment	Population development level	1. Population (million people)	−	0.8100
		2. Urbanization rate (%)	+	0.2100
	Economic development level 1. Gross regional product (in 100 million yuan)		+	0.2280
		2. GDP per capita (10,000 yuan/person)	+	0.1131
		3. Share of the secondary industry (%)	−	0.4710
		4. Share of the tertiary industry (%)	+	0.2280
	Resource and environment development level	1. Share of nonfossil energy (%)	+	0.1100
		2. Energy consumption intensity (t/10000)	−	0.2100
		3. Carbon emission intensity (in 10,000 yuan)	−	0.4100
		4. SO_2_ emissions (million *t*)	−	0.1000
		5. CO_2_ emissions (million *t*)	−	0.1000
		6. Forest coverage rate (%)	+	0.1100

**Table 2:** Determination criteria and classification system for the degree of coordinated development of regional population, economy, and resources and environment.

Classification	Degree of coordinated development	Level 1 criteria	Level 2 criteria
Interval of coordinated development	0.9∼1	Superior coordination	① When *m* = min{/(x),*g*(*y*),h(z)}, it is a lagged type, similar to *m* = /(x), which is population lagged type. ② When/(x) = *g*(*y*) = *h*(z), it is a synchronous type for population, economy, and environment
	0.80∼0.89	Good coordination	
	0.70∼0.79	Moderate coordination	
Interval of transitional reconciliation	0.60∼0.69	Basically coordinated	
	0.50∼0.59	Barely coordinated	
	0.40∼0.49	On the verge of dysfunction and decline	
Interval of dysfunction and decline	0.30∼0.39	Mild dysfunction and decline	
	0.20〜0.29	Moderate dysfunction and decline	
	0.10∼0.19	Severe dysregulation and decline	
	0∼0.09	Extremely severe dysfunction and decline	

**Table 3:** Settings of the relevant parameters in the stage mode.

Classification	Degree of coordinated development	Level 1 criteria	Level 2 criteria
Interval of coordinated development	0.9∼1	Superior coordination	① When *m* = min{/(*x*), *g*(*y*), *h*(*z*)}, it is a lagged type, similar to *m* = /(*x*), which is population lagged type. ② When/(*x*) = *g*(*y*) = *h*(*z*), it is a synchronous type for population, economy, and environment
	0.80∼0.89	Good coordination	
	0.70∼0.79	Moderate coordination	
Interval of transitional reconciliation	0.60∼0.69	Basically coordinated	
	0.50∼0.59	Barely coordinated	
	0.40∼0.49	On the verge of dysfunction and decline	
Interval of dysfunction and decline	0.30∼0.39	Mild dysfunction and decline	
	0.20〜0.29	Moderate dysfunction and decline	
	0.10∼0.19	Severe dysregulation and decline	
	0∼0.09	Extremely severe dysfunction and decline	

Note: low, medium, and high stand for high growth rate, medium growth rate, and low growth rate, respectively. The parameters of the province are set based on the “12th Five-Year Plan”; the plan refers to the leapfrog plan of the key advantageous industries in the province, the establishment industry development plan of the province, the establishment industry development plan of the province in 2013, and the studies by scholars such as Lin Fude and Fu Jiafeng.

## Data Availability

The labeled data set used to support the findings of this study is available from the corresponding author upon request.

## References

[1] Biao M. (2017). Literature review on land carrying capacity of the coordinated development of population, resources, environment and economy. *AIP Conference Proceedings*.

[2] Zhu J. (2019). Population,resources,the environment and sustainable development. *Meteorological and Environmental Research*.

[3] Binglin L., Shengnan J., Minghui X., Yun X. (2019). Coordinated development of population, economy and environment system and diagnosis of its obstacle factors in nanjing. *Meteorological and Environmental Research*.

[4] Zhang L.-G., Hui L., Hua C., Liu Z. (2019). [Analysis on the coordinated development of ecology-economy-society in coal resource cities: a case study of huainan, China]. *Ying yong sheng tai xue bao = The journal of applied ecology*.

[5] Gremillion K. J., Shane miller D. (2019). From Colonization to Domestication: Population, Environment, and the Origins of Agriculture in Eastern north america. salt lake city. *Antiquity*.

[6] Cui X., Fang C., Liu H., Liu X. (2019). Assessing sustainability of urbanization by a coordinated development index for an urbanization-resources-environment complex system: a case study of jing-jin-ji region, China. *Ecological Indicators*.

[7] Chen C., Ding Z., Kang J., Yanfei L. I. (2017). The coupling coordinated development of urbanization and resource-environment system in henan. *Journal of Henan University*.

[8] Zhu S. L., Wang H., Wang W. T., Zhou X., Liu Y. H. (2017). Status of coordinated development between regional low-carbon economy and spatial land-use pattern in the 12∼(th) five year plan. *China Population,Resources and Environment*.

[9] Nie X., Zhang T., Zhu L. I., Feng F. (2018). Ecological civilization evaluation and coordinated development between environment,economy and society in hubei. *Journal of Shanxi Normal University (Philosophy and Social Sciences edition)*.

[10] Sun Q., Zhang X., Zhang H., Niu H. (2018). Coordinated development of a coupled social economy and resource environment system: a case study in henan province, China. *Environment, Development and Sustainability*.

[11] Wei H. E. (2019). Relationship between economic development and environmental pollution in resource-based city——a case study of hengyang, hunan province. *Territory & Natural Resources Study*.

[12] Wang F., University H. N. (2017). Evaluation of coordinated development between water resources and ecological environment of wanjiang city belt. *Pearl River*.

[13] Zhang H., Zhu Z., Fan Y. (2018). The impact of environmental regulation on the coordinated development of environment and economy in China. *Natural Hazards*.

[14] Jiang X. J., Yang Q. S., Geng Q. G., Wang X. Y., Liu J. (2019). Spatial-temporal differentiation and driving mechanism of coordinated development of ecological-economic-society systems in the yangtze river economic belt. *Resources and Environment in the Yangtze Basin*.

[15] Xiao-Yong Y. U., Zhang L. P., Chen X. C., Yang K., Huang Y. Q. (2018). Analysis of coupling and coordinated development between water resources and social economy in hubei province. *Resources and Environment in the Yangtze Basin*.

